# 

**Published:** 1901-08

**Authors:** 


### Contributors to New Series Volume XLI.

### (Whole Number Volume LVII.)

### August, 1901–July, 1902.

| | |
|---|---|
| Beahan, A. L. | Canandaigua |
| Bennett, A. G. | Buffalo |
| Blackham, George E. | Dunkirk |
| Brown, George L. | Buffalo |
| Chanthard, A. | Paris |
| Clinton, Marshall | Buffalo |
| Conklin, William L. | Rochester |
| Cott, George F. | Buffalo |
| Curtis, F. C. | Albany |
| Daggett, Byron H. | Buffalo |
| Diehl, Alfred E. | Buffalo |
| Dowd, J. Henry | Buffalo |
| Durrent, J. A. | Marysville, Wash. |
| Earle, Frank B. | Chicago |
| Evans, William James | New York |
| Flagg, John D. | Buffalo |
| Floeckinger, F. C. | La Grange, Texas |
| Forsyth, Edgar A. | Buffalo |
| Fronczak, Francis E. | Buffalo |
| Frye, Maud J. | Buffalo |
| Gardner, J. A. | Buffalo |
| Gaylord, Harvey R. | Buffalo |
| Gram, Franklin C. | Buffalo |
| Grant, John H | Buffalo |
| Greene, Walter David | Buffalo |
| Grosvenor, J. W. | Buffalo |
| Greenleaf, Clarence A. | Rochester |
| Guiteras, Ramon | New York |
| Hamann, C. A. | Cleveland |
| Hanley, L. G. | Buffalo |
| Harrington, Devillo W. | Buffalo |
| Hartwig, Marcell | Buffalo |
| Hauenstein, John | Buffalo |
| Heuman, Charles C. | Brooklyn |
| Heffron, John L. | Syracuse |
| Himmelsbach, G. A. | Buffalo |
| Hobbs, A. T. | Guelph, Ont. |
| Hopkins, Henry Reed | Buffalo |
| Howe, Lucien | Buffalo |

Hubbell, Alvin A. . . . . . . . . . . . . . . . . . . . . . . . Buffalo
Jeffries, F. M. . . . . . . . . . . . . . . . . . . . . . New York
Jones, William B. . . . . . . . . . . . . . . . . . . . . . . Rochester
King, James E. . . . . . . . . . . . . . . . . . . . . . . . . Buffalo
Krauss, William C. . . . . . . . . . . . . . . . . . . . . . . Buffalo
La Garde, Major L. A. . . . . . . . . . . . . . . . . . . Washington, D. C.
LeBreton, Prescott . . . . . . . . . . . . . . . . . . . . . . . Buffalo
Levien, Henry . . . . . . . . . . . . . . . . . . . . . . . Liberty, N. Y.
Lewi, Maurice J. . . . . . . . . . . . . . . . . . . . . . . . New York
Lewis, F. Park . . . . . . . . . . . . . . . . . . . . . . . . . Buffalo
Lyon, Irving P. . . . . . . . . . . . . . . . . . . . . . . . . Buffalo
Mann, Matthew D. . . . . . . . . . . . . . . . . . . . . . . . Buffalo
Matzinger, Herman G. . . . . . . . . . . . . . . . . . . . . . Buffalo
Mays, Thomas J. . . . . . . . . . . . . . . . . . . . . . . Philadelphia
McGuire, Frank W. . . . . . . . . . . . . . . . . . . . . . . . Buffalo
McMichael, George H. . . . . . . . . . . . . . . . . . . . . . Buffalo
McMurtry, Lewis S. . . . . . . . . . . . . . . . . . . . . . . Louisville
Mynter, Herman . . . . . . . . . . . . . . . . . . . . . . . . . Buffalo
Park, Roswell . . . . . . . . . . . . . . . . . . . . . . . . . . Buffalo
Parmenter, John . . . . . . . . . . . . . . . . . . . . . . . . . Buffalo
Peterson, Frederick . . . . . . . . . . . . . . . . . . . . . . New York
Phelps, A. M. . . . . . . . . . . . . . . . . . . . . . . . . . New York
Phelps, William C. . . . . . . . . . . . . . . . . . . . . . . . Buffalo
Potter, Julius H. . . . . . . . . . . . . . . . . . . . . . . . Buffalo
Potter, William Warren . . . . . . . . . . . . . . . . . . . . . Buffalo
Pryor, W. R. . . . . . . . . . . . . . . . . . . . . . . . . . New York
Putnam, James Wright . . . . . . . . . . . . . . . . . . . . . . Buffalo
Renner, W. Scott . . . . . . . . . . . . . . . . . . . . . . . . Buffalo
Rochester, DeLancey . . . . . . . . . . . . . . . . . . . . . . Buffalo
Sargent, George W. . . . . . . . . . . . . . . . . . . . . Seneca Castle
Sloan, W. Harpur . . . . . . . . . . . . . . . . . . . . . . Philadelphia
Smith, Eugene A. . . . . . . . . . . . . . . . . . . . . . . . . Buffalo
Snow, Sargent F. . . . . . . . . . . . . . . . . . . . . . . . Syracuse
Spratling, William P. . . . . . . . . . . . . . . . . . . . Sonyea, N. Y.
Van der Beek, C. A. . . . . . . . . . . . . . . . . . . . . . . Rochester
Wende, Grover W. . . . . . . . . . . . . . . . . . . . . . . . . Buffalo
Wilson, Nelson W. . . . . . . . . . . . . . . . . . . . . . . . Buffalo
Wynkoop, Edward Judson . . . . . . . . . . . . . . . . . . . . . Syracuse
Young, A. A . . . . . . . . . . . . . . . . . . . . . . . . Newark, N. Y.

Alumni Association University of Buffalo
Buffalo Academy of Medicine.
Buffalo Ophthalmological Club.
Medical Society of the County of Erie.
Medical Association of Central New York.
Physicians' League of Buffalo.
Roswell Park Medical Club.

### BOOKS RECEIVED.

Essentials of Refraction and of Diseases of the Eye. By Edward Jackson, A. M , M. D., Emeritus Professor of Diseases of the Eye in the Philadelphia Polyclinic. Third edition, revised and enlarged. Duodecimo, 261 pages, 82 illustrations. Philadelphia and London; W. B. Saunders & Co. 1901. [Price, cloth, $1.00 net.]

Practical Surgery: A Work for the General Practitioner. By Nicholas Senn, M. D., Ph. D., LL. D., Professor of Surgery, Rush Medical College, Chicago. Handsome octavo volume of 1133 pages, with 650 illustrations, many in colors. Philadelphia and London: W. B. Saunders & Co. 1901. [Price, cloth, $6.00 net.]

The Hygiene of Transmissible Diseases: Their Causation, Modes of Dis semination and Methods of Prevention. By A. C. Abbott, M. D., Professor of Hygiene and Bacteriology, University of Pennsylvania. Third edition, revised and enlarged. Octavo, 351 pages, with numerous illustrations. Philadelphia and London. W. B. Saunders & Co. 1901. [Price, cloth, $2.50 net.]

Diseases of the Intestines. By Dr. I. Boas, Specialist for Gastro-Intestinal Diseases in Berlin. Authorised translation from the first German edition, with special additions by Seymour Basch, M. D., New York City. Octavo, pp. 574, 47 illustrations. New York: D. Appleton & Co. 1901 [Price, cloth, $5.00; sheep, $6.00.]

The Johns Hopkins Hospital Reports. Volume X., Nos. 1 and 2. Baltimore: The Johns Hopkins Press. 1901.

Transactions of the Southern Surgical and Gynecological Association. Volume XIII. Thirteenth annual session, held at Atlanta, Ga., November 13, 14 and 15, 1901. Secretary, Dr. W. D. Haggard Jr., Nashville. Published by the Association. Philadelphia: W. J. Dornan, Printer. 1901.

Bulletin of the Buffalo Society of Natural Sciences. Guide to the Geology and Paleontology of Niagara Falls and Vicinity. By A. W. Grabau. Albany: University of the State of New York. 1901.

